# Clarifications on the article “knowledge, attitudes, and perception of the role of the media regarding COVID-19 in medical students from a peruvian university”

**DOI:** 10.17843/rpmesp.2022.392.11071

**Published:** 2022-06-30

**Authors:** José A. Gonzales-Zamora

**Affiliations:** 1 División de Enfermedades Infecciosas, Departamento de Medicina. University of Miami, Miller School of Medicine. Miami, Florida, USA. Miami University División de Enfermedades Infecciosas, Departamento de Medicina University of Miami Miller School of Medicine Miami, Florida USA; 2 Peruvian American Medical Society, Albuquerque, New Mexico, USA. Peruvian American Medical Society Albuquerque New Mexico USA

To the editor. I have read with great interest the article published in volume 39 of your journal, which evaluates the level of knowledge and attitudes of medical students about COVID-19, in addition to their perception of the role of the media and social networks, for which a virtual survey was used ^1^. The findings are very interesting; however, I find some contradictions in the results. In the abstract of the article, it is mentioned that “32% did not know that during the first five days of illness, serological tests are preferred for diagnosing COVID-19 over molecular tests”. This statement is incorrect, since in the first days of illness molecular tests are preferred due to the low sensitivity of serological tests ^2^. When reviewing the content of the article, it was observed that in the results section it is mentioned that “31% (n=38) did not know that in the first 14 days of illness molecular tests are preferred over serological tests” ^1^. It can be inferred that what was stated in the abstract was a drafting or printing error. However, it could also be noted that in Table 2 of the manuscript (section number 16) the students were asked about the diagnostic superiority of molecular tests in the first five days of illness, being this period of time completely different from the 14 days mentioned in the text ^1^. This lack of uniformity in the presentation of results generates a great deal of confusion, requiring clarification by the authors, since it is one of the main findings of their research. 

Likewise, it was noted that one of the questions on knowledge made reference to herd immunity, asking the question “Do you consider that Peru has already reached herd immunity?”. We have to be careful with this concept, since it is a phenomenon that requires the population to develop a high rate of acquired immunity (by vaccination or natural infection) in order to stop the transmission of COVID-19; however, there are many factors that make this unfeasible ^3^. Available vaccines have shown great efficacy in preventing severe disease and mortality; however, their efficacy is not very high at preventing infection and, therefore, they could not completely stop community transmission ^4^. In addition, the appearance of variants such as omicron allow the development of infection and symptomatic disease, despite the presence of antibodies, due to mutations in the spike protein that allow evading this defense mechanism ^5^. Finally, protection provided by antibodies is not very durable, and the population may be exposed to a new infection several months after receiving the vaccine or become infected by SARS-CoV-2 ^6^. Therefore, I consider that asking whether Peru has reached herd immunity would be an inappropriate question to measure the level of knowledge, since it would be based on a concept that is inapplicable to COVID-19. In view of the above, I believe it would be pertinent to clarify these details for a better understanding of the findings.
