# Supplementary material for: Clarifications on the article “knowledge, attitudes, and perception of the role of the media regarding COVID-19 in medical students from a peruvian university”
Source: Rev Peru Med Exp Salud Publica. 2022 Jun 30;39(2):249–50. doi: 10.17843/rpmesp.2022.392.11071 (PMC11397589; doi:10.17843/rpmesp.2022.392.11071)
Supplement: Translation

# Translation: Precisiones sobre el artículo “Conocimientos, actitudes y percepción sobre el rol de los medios de comunicación respecto a la COVID-19 en estudiantes de medicina de una universidad peruana”

**Authors:** José A. Gonzales-Zamora

**Affiliations:** 1 Division of Infectious Diseases, Department of Medicine. University of Miami, Miller School of Medicine. Miami, Florida, USA.; 2 Peruvian American Medical Society, Albuquerque, New Mexico, USA.

Sr. Editor. He leído con gran interés el articulo publicado en el volumen 39 de su revista, en el cual se evalúa el nivel de conocimientos y actitudes de los estudiantes de Medicina sobre la COVID-19, además de su percepción sobre el rol de los medios de comunicación y las redes sociales, para lo cual se empleó una encuesta virtual ^1^. Los hallazgos son muy interesantes; sin embargo, encuentro ciertas contradicciones en los resultados. En el resumen del artículo se menciona que «el 32% no sabía que, en los primeros cinco días de la enfermedad, las pruebas serológicas son preferibles para diagnosticar la COVID-19, comparadas con las pruebas moleculares». Esta aseveración es incorrecta, ya que en los primeros días de la enfermedad se prefieren las pruebas moleculares debido a la baja sensibilidad de las pruebas serológicas ^2^. Al revisar el contenido del artículo se pudo observar que en la sección de resultados se menciona que «el 31% (n=38) no sabía que en los primeros 14 días de enfermedad las pruebas serológicas no son preferibles en comparación con las moleculares» ^1^. Se puede colegir que lo manifestado en el resumen se trataría de un error de redacción o impresión. Sin embargo, también se pudo notar que en la Tabla 2 del manuscrito (acápite número 16) se preguntó a los alumnos sobre la superioridad diagnóstica de las pruebas moleculares en los primeros cinco días de enfermedad, siendo este periodo de tiempo completamente distinto a los 14 días mencionado en el texto ^1^. Esta falta de uniformidad en la presentación de resultados genera muchísima confusión, requiriendo una aclaración por parte de los autores, ya que trata de uno de los principales hallazgos de su investigación. 

Asimismo, se observó que una de las preguntas sobre conocimiento hizo referencia a la inmunidad de rebaño, formulándose la pregunta «¿Considera usted que el Perú ya ha llegado a la inmunidad de rebaño?». Tenemos que ser cuidadosos con este concepto, ya que es un fenómeno que requiere que la población desarrolle una tasa de inmunidad adquirida (por vacunación o infección natural) tan alta que haga posible detener la transmisión de la COVID-19; sin embargo, existen muchos factores que lo hacen inviable ^3^. Las vacunas disponibles han mostrado una gran eficacia para prevenir enfermedad severa y mortalidad; no obstante, su eficacia no es muy alta para prevenir infección y, por ende, no podrían bloquear completamente la transmisión comunitaria ^4^. Además, la aparición de variantes como ómicron hacen posible el desarrollo de infección y enfermedad sintomática, a pesar de la presencia de anticuerpos debido a mutaciones en la proteína spike que permiten evadir este mecanismo de defensa ^5^. Por último, la protección obtenida por anticuerpos no es muy duradera, pudiendo la población quedar expuesta a una nueva infección luego de varios meses de recibir la vacuna o infectarse por SARS-CoV-2 ^6^. Por lo tanto, considero que preguntar si el Perú ha llegado a la inmunidad de rebaño, sería una pregunta inapropiada para medir el nivel de conocimiento, ya que partiría de un concepto inaplicable a COVID-19. Por lo expuesto, creo que sería pertinente aclarar estos detalles para una mejor comprensión de los hallazgos.
